# Microsatellite instability in Japanese female patients with triple-negative breast cancer

**DOI:** 10.1007/s12282-019-01043-5

**Published:** 2020-01-06

**Authors:** Kanako Kurata, Makoto Kubo, Masaya Kai, Hitomi Mori, Hitomi Kawaji, Kazuhisa Kaneshiro, Mai Yamada, Reiki Nishimura, Tomofumi Osako, Nobuyuki Arima, Masayuki Okido, Yoshinao Oda, Masafumi Nakamura

**Affiliations:** 1 Department of Surgery and Oncology, Graduate School of Medical Sciences, Kyushu University, 3-1-1 Maidashi, Higashi-ku, Fukuoka, 812-8582 Japan; 2 Breast Center, Kumamoto Shinto General Hospital, 3-2-65 Oe, Chuo-ku, Kumamoto, 862-8655 Japan; 3 Department of Pathology, Kumamoto Shinto General Hospital, 3-2-65 Oe, Chuo-ku, Kumamoto, 862-8655 Japan; 4 Department of Surgery, Hamanomachi Hospital, 3-3-1 Nagahama, Chuo-ku, Fukuoka, 810-8539 Japan; 5 Department of Anatomic Pathology, Graduate School of Medical Sciences, Kyushu University, 3-1-1 Maidashi, Higashi-ku, Fukuoka, 812-8582 Japan

**Keywords:** Microsatellite instability, Triple-negative breast cancer, Biomarker, PD-1/PD-L1 blockade, Immune checkpoint inhibitor

## Abstract

**Background:**

It is important to identify biomarkers for triple-negative breast cancers (TNBCs). Recently, pembrolizumab, an immune checkpoint inhibitor (ICI) for programmed cell death 1 (PD-1), was approved as a treatment strategy for unresectable or metastatic tumor with high-frequency microsatellite instability (MSI-H) or mismatch repair deficiency, such as malignant melanoma, non-small cell lung cancer, renal cell cancer and urothelial cancer. In addition, results from clinical trials suggested that ICI was a promising treatment for TNBCs with accumulated mutations. However, the frequency of MSI in Japanese TNBCs still remains unclear. We aimed to analyze the presence of MSI-H in TNBCs as a biomarker for ICI therapy.

**Methods:**

In this study, we retrospectively evaluated the MSI of 228 TNBCs using an innovative method, MSI Analysis System Version 1.2 (Promega), consisting of 5 microsatellite markers: BAT-26, NR-21, BAT-25, MONO-27 and NR-24 without a normal tissue control.

**Results:**

Among 228 tumors, 222 (97.4%) were microsatellite stable, 4 (1.7%) low-frequency MSI and 2 (0.9%) MSI-H, respectively. Two MSI-H tumors were potentially aggressive pathologically as indicated by nuclear grade 3 and high Ki-67 (> 30%), and were classified as basal-like and non-BRCA-like, but were not consistent regarding tumor-infiltrating lymphocytes, CD8 and PD-L1 expression.

**Conclusions:**

Although we found that MSI-H was uncommon (0.9%) in TNBCs, potential targets for ICIs exist in TNBCs. Therefore, MSI-H breast cancer patients should be picked up using not only conventional methods but also platforms for comprehensive genomic profiling.

## Introduction

Triple-negative breast cancers (TNBCs) are defined as tumors that lack the expression of estrogen receptor (ER), progesterone receptor (PR), and human epidermal growth factor receptor 2 (HER2). Therefore, chemotherapy remains the mainstay of systemic treatment for patients with TNBCs, because they cannot benefit from endocrine therapy or trastuzumab [1]. TNBC is, in general, a high-grade and aggressive disease with a high rate of distant metastasis, and is correlated with a poorer outcome compared with other breast cancer subtypes. To improve the therapeutic effects and prognosis for TNBCs, it is necessary to establish new treatment strategies and specific biomarkers.

Microsatellite instability (MSI) is a phenotype resulting from a defect in mismatch repair (MMR) genes. MMR deficiency (dMMR) is present in various cancers, including those of the colorectum, uterus, stomach, biliary tract, pancreas, ovary, prostate, and small intestine [2–5]. Lynch syndrome (LS), also known as hereditary nonpolyposis colorectal cancer, is a common autosomal dominant syndrome characterized by early age at onset, neoplastic lesions, and MSI. Tumors with MSI account for approximately 15% of all colorectal cancers [6]. dMMR colorectal cancers are more responsive to programmed cell death 1 (PD-1)/programmed death-ligand 1 (PD-L1) blockade than MMR-proficient (pMMR) colorectal cancers [2]. The US Food and Drug Administration (FDA) approved anti-PD-1 immune checkpoint inhibitor (ICI), pembrolizumab, for the treatment of adult and pediatric patients with unresectable or metastatic, high-frequency MSI (MSI-H) or dMMR solid tumors in May 2017. These are the first gene level biomarkers for anti-PD-1 ICIs, which were approved in Japan in December 2018.

MSI and dMMR were uncommon in breast cancer [7, 8]. Therefore, MSI-H breast cancer patients could be picked up using not only conventional methods but also platforms for comprehensive genomic profiling. FoundationOne CDx is the first FDA-approved broad companion diagnostic (CDx) for solid tumors, including MSI and tumor mutational burden (TMB) to help inform immunotherapy decisions [9]. Also, FDA granted Breakthrough Device Designation for its pan-cancer assay, TruSight Oncology 500 panel (Illumina, San Diego, CA, USA), in January 2019 [10], which gave oncologists information on MSI and TMB with 500 genes. Hempelmann et al. [11] demonstrated that next-generation sequencing (NGS) methods had superior sensitivity and offered advantage over the widely used 5-marker MSI polymerase chain reaction (PCR) in prostate cancer. However, according to ‘Bethesda guidelines’ for colorectal cancers, a panel with five poly-A mononucleotide repeats (BAT-25, BAT-26, NR-21, NR-24, MONO-27) is considered the current standard because of its higher specificity and sensitivity. ESMO strongly recommends that the first line of molecular analysis is represented by PCR for MSI testing in the framework of immunotherapy, but very strongly that an NGS represents an alternative molecular test to assess MSI [12]. Although the main advantages of NGS method are simultaneously represented by the analyses on MSI and TMB, we have to wait for a while until they became the next CDx.

Some tumors with genomic instability respond well to PD-1/PD-L1 blockade, suggesting that this is a promising target for some refractory breast cancers. However, a previous study reported that MSI and dMMR were infrequent in breast cancer [7]. Therefore, this study analyzed the presence of MSI in Japanese female patients with TNBCs as a biomarker for ICIs and confirmed fundamental data on the frequency of MSI status in Japan.

## Materials and methods

### Patients

This study included 228 patients with primary TNBC who underwent resection without neoadjuvant chemotherapy at Kyushu University Hospital (Fukuoka, Japan), Hamanomachi Hospital (Fukuoka, Japan) or Kumamoto City Hospital (Kumamoto, Japan) between January 2004 and December 2014. Elucidation of tumor subtypes was determined by immunohistochemistry (IHC) staining of surgically resected tissues. Classification of ER or PR positivity was defined as ≥ 1% of tumor cells staining positive for ER or PR. Cancer specimens were defined as HER2 positive when HER2 IHC staining was scored as 3+ according to the standard criteria [13, 14], or when HER2 gene amplification was detected using fluorescence spectroscopy with in situ hybridization. The current study conformed to the principles of the Declaration of Helsinki and was approved by the Institutional Review Board of Kyushu University Hospital (No. 30-231).

### Analysis of microsatellite instability

Surgical specimens were used for MSI analysis. Genomic DNA was extracted from FFPE using a QIAamp DNA FFPE Tissue Kit (QIAGEN, Hilden, Germany). The tumor content required for the analysis was 40% or more, and if it was less than 40%, genomic DNA was extracted by macrodissection. MSI analysis was performed using the MSI Analysis System Version 1.2 (Promega, Madison, WI, USA) with the following 5 microsatellite markers: BAT-26, NR-21, BAT-25, MONO-27 and NR-24 according to the quasi-monomorphic variation range (QMVR) method without paired normal DNA reported previously [15]. Previous report showed that the sensitivity and specificity of this QMVR method were concordant with the standard method using tumor DNA plus paired normal DNA [16]. Tumors exhibiting markers outside the corresponding QMVR were defined as MSI. We classified the tumors as MSI-H if two or more of the five markers showed MSI and low-frequency MSI (MSI-L) if any one marker showed MSI. Microsatellite stable (MSS) tumors were characterized by the absence of MSI by all 5 markers.

### Multiplex ligation-dependent probe amplification (MLPA) method

Also, surgical specimens were used for MLPA analysis to determine the presence of BRCAness, as previously reported [17]. MLPA was undertaken to determine the relative copy number of various DNA sequences using the MLPA probe mix containing 38 target probes, which covered the most important genomic regions of the BRCA1-like classifier based on specific aberrations of BRCA1-mutated breast cancer compared with sporadic tumors by array comparative genomic hybridization. The relative copy number ratio of each sample was compared using Human Genomic DNA (Promega, Madison, WI, USA) as a reference sample. BRCAness scores were calculated with the relative copy number ratios of various DNA sequences. Each sample was analyzed twice by researchers and the mean score was used for the analysis. If the BRCAness score of a sample was ≥ 0.5, it was classified as BRCAness and if the score was < 0.5, the sample was classified as being non-BRCAness.

### Evaluation of tumor-infiltrating lymphocytes (TILs)

TILs were assessed in hematoxylin and eosin (HE)-stained sections, following guidelines published by the International TILs Working Group to standardize TILs evaluation [18, 19]. Cases were defined as TILs-High for ≥ 50% stromal TILs and TILs-low for < 50% stromal TILs [20].

### IHC staining

Epidermal growth factor receptor (EGFR) primary antibody (monoclonal mouse, clone DAK-H1-WT, Dako, Glostrup, Denmark), cytokeratin 5/6 (CK5/6) primary antibody (monoclonal mouse, clone D5/16 B4, Dako), anti-PD-L1 antibody (monoclonal rabbit, E1L3N; Cell Signaling Technology, Beverly, MA), and anti-CD8 antibody (monoclonal mouse, C8/144B; Nichirei Bioscience Inc., Tokyo, Japan) were used with a Ventana Discovery XT automated stainer (Ventana Medical Systems, Tucson, AZ, USA) with proprietary reagents according to the manufacturer’s protocol. A basal-like phenotype was defined as positive for EGFR and/or CK5/6. PD-L1 positivity was defined as PD-L1 expression in ≥ 1% of tumor cells [20]. CD8-positive T cells were counted separately by their localization such as intratumoral or stromal with a microscope field at 200× magnification (0.00625 mm^2^). Five areas with the most abundant infiltration were selected, and the average count was calculated. The results were interpreted as positive when there were more than or equal to 30 cells per 0.0625 mm^2^ in intratumoral or stromal area [21].

## Results

Age at diagnosis, tumor size, nodal status, pathological stage, histological characteristics, presence of TILs and CD8-positive T cells, PD-L1 expression, the interaction between PD-L1 and TILs, basal-like status, BRCAness status, and MSI status of 228 TNBC patients are listed in Table 1. The mean age of patients was 59 years (range: 30–89) and all were women. Of 228 tumors, 132 (57.9%) were T1 tumors and 154 (67.5%) were node-negative tumors; 151 (66.2%) and 152 (66.7%) were tumors with nuclear grade (NG) 3 and with high Ki-67 (> 30%), respectively; 99 (43.4%) were classified as TILs-High, 112 (49.1%) had CD8-positive T cells, 90 (39.5%) had PD-L1 expression on tumor cells, 203 (89.0%) were tumors with basal-like features, and 148 (64.9%) had BRCAness. IHC staining was not performed for 13 cases. Among 228 tumors, 222 (97.4%) were MSS, 4 (1.7%) were MSI-L, and 2 (0.9%) were MSI-H.

**Table 1:** Clinicopathologic characteristics of patients with TNBC

	Number of patients	
	*N* = 228	
Age at diagnosis (y), mean (range)	59	(30–89)
Tumor size		
T1a/b (≤ 1 cm)	19	(8.3%)
T1c (> 1 cm, ≤ 2 cm)	113	(49.6%)
T2 (> 2 cm, ≤ 5 cm)	89	(39.0%)
T3 (> 5 cm)	6	(2.6%)
T4	1	(0.4%)
Nodal status		
N0	154	(67.5%)
N1 (1–3)	56	(24.6%)
N2 (4–9)	11	(4.8%)
N3 (≥ 10)	7	(3.0%)
Pathological stage		
I	98	(43.0%)
II	109	(47.8%)
III	21	(9.2%)
Nuclear grade		
1/2	70	(30.7%)
3	151	(66.2%)
Unknown	7	(3.1%)
Ki-67		
≤ 30%	40	(17.5%)
> 30%	152	(66.7%)
Unknown	36	(15.8%)
TILs		
High	99	(43.4%)
Low	116	(50.9%)
N/A	13	(5.7%)
CD8		
+	112	(49.1%)
−	103	(45.2%)
N/A	13	(5.7%)
PD-L1		
+	90	(39.5%)
−	125	(54.8%)
N/A	13	(5.7%)
PD-L1 * TILs		
PD-L1+/TILs-High	74	(32.5%)
PD-L1−/TILs-High	25	(11.0%)
PD-L1+/TILs-Low	16	(7.0%)
PD-L1−/TILs-Low	100	(43.9%)
N/A	13	(5.7%)
Basal-like status		
+	203	(89.0%)
−	23	(10.1%)
N/A	2	(0.9%)
BRCAness status		
+	148	(64.9%)
−	78	(34.2%)
N/A	2	(0.9%)
MSI status		
MSS	222	(97.4%)
MSI-L	4	(1.7%)
MSI-H	2	(0.9%)

N/A, not available; y, years; PD-L1, programmed death-ligand 1; TILs, tumor-infiltrating lymphocytes; *, interaction; MSS, microsatellite stable; MSI-L, low-frequency microsatellite instability; MSI-H, high-frequency microsatellite instability

**Fig. 1:**
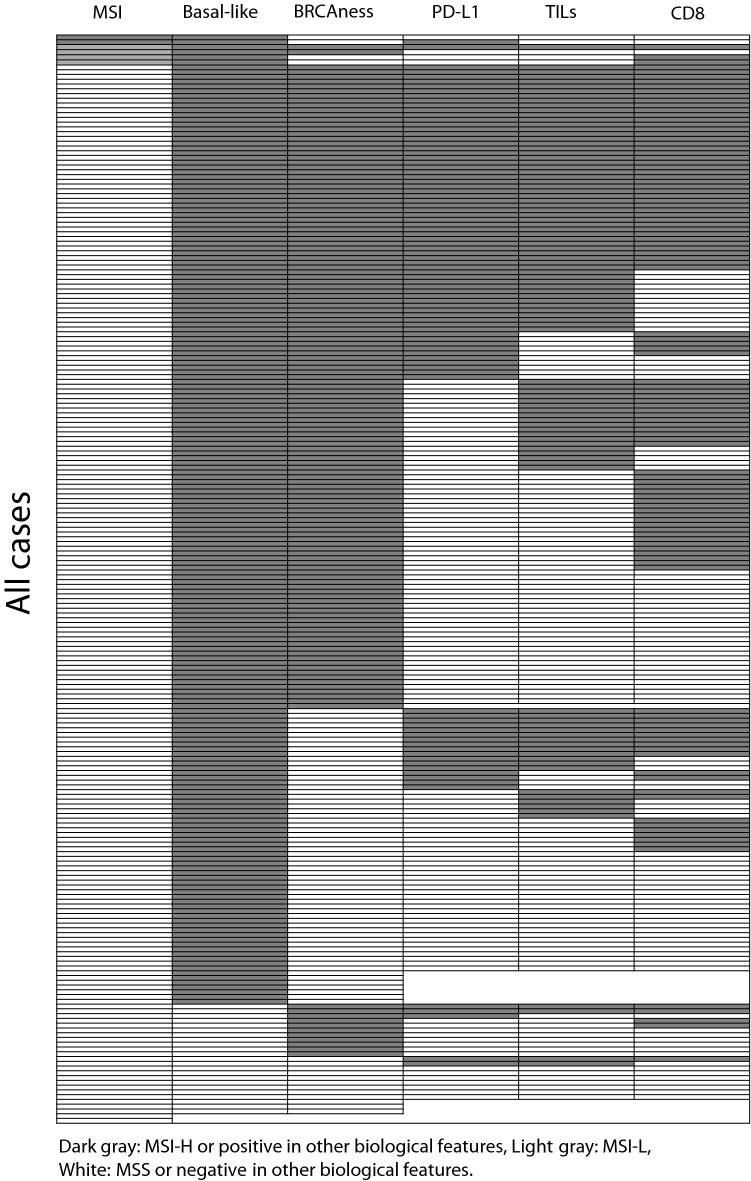
Comprehensive datasheet regarding the association between MSI and other biological features (basal-like, BRCAness, PD-L1, TILs and CD8) in all cases. Each row represents one case. Dark gray: MSI-H, BRCAness, PD-L1 positive, TILs-High, CD8-positive, light gray: MSI-L, white: MSS, non-BRCAness, PD-L1 negative, TILs-Low, CD8-negative

The heatmap of the association between MSI status, basal-like status, BRCAness status, PD-L1 expression, presence of TILs, and that of CD8-positive T cells in all patients is shown in Fig. 1. Forty-four cases had all positive and 7 had all negative in the five statuses excluding MSI. As is demonstrated in Table 1 and Fig. 1, 74 (82.2%) of 90 PD-L1 positive cases showed TILs-High. Conversely, only 25 (20.0%) of 125 PD-L1 negative cases showed TILs-High.

**Fig. 2:**
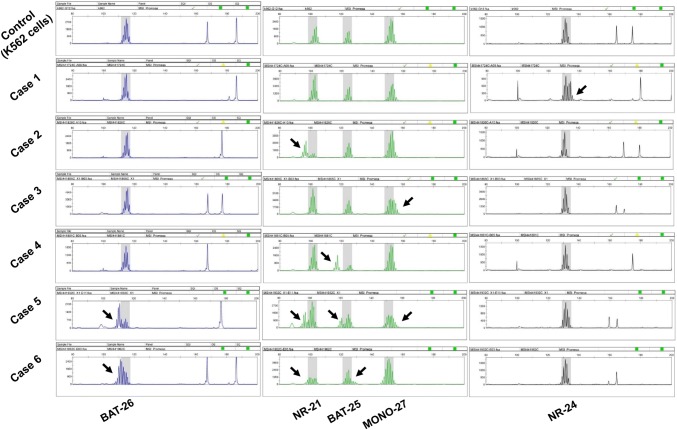
Microsatellite instability analysis of MSI-H and MSI-L tumors. Electropherograms show the peak of fluorescein-labeled loci BAT26, NR21, BAT25, MONO27 and NR24. Instability is indicated when a peak exceeds the control width

**Fig. 3:**
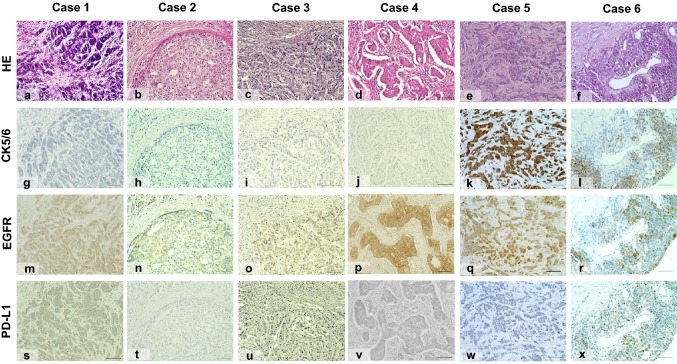
Microscopic findings of MSI-L (Case 1–4) and MSI-H (Case 5, 6) tumors (magnification; ×200, Bar; 100 μm). HE staining shows tumors in Cases 1–6 (**a**–**f**). IHC images show CK5/6 expressed positive in case 5, 6 (**k**, **l**), but not in Case 1–4 (**g**–**j**). EGFR was expressed as positive in Cases 1–6 (**m**–**r**). PD-L1 was expressed as positive in Case 1 and 6 (**s**, **x**), but not in Case 2–5 (**t**–**w**)

The two MSI-H tumors had the following three common instability markers: BAT-26, NR21 and BAT-25 (Table 2 and Fig. 2). Of these tumors, one showed T1N0 and another T2N0. Both had aggressive features including NG 3 (Table 2 and Fig. 3e, f) and high Ki-67 (> 30%), and were classified as basal-like and non-BRCAness (Table 2 and Fig. 3k, l, q, r). Only one of the two tumors expressed PD-L1 (Table 2 and Fig. 3w, x) and, they had TILs-Low and CD8-negative T cells in their microenvironment (Table 2). Each of the 4 MSI-L tumors had a different instability marker (Table 2 and Fig. 2). Of the 4 tumors, one had TILs-High and the others had TILs-Low (Table 2 and Fig. 3a–d). All were classified as basal-like and 2 were BRCAness (Table 2 and Fig. 3g–j, m–p). Only one tumor expressed PD-L1 (Table 2 and Fig. 3s–v).

**Table 2:** Clinicopathologic characteristics of tumors with microsatellite instability

	Case number					
	1	2	3	4	5	6
Age	67	73	61	56	80	74
pTN classification	T3N0	T2N1	T1N0	T2N0	T1N0	T2N0
Nuclear grade	3	2	2	1	3	3
Ki67 (%)	50	18	37	8	54	92
TILs	High	Low	Low	Low	Low	Low
CD8	+	−	+	+	−	−
PD-L1	+	−	−	−	−	+
Basal-like status	+	+	+	+	+	+
BRCAness status	+	+	−	−	−	−
MSI	Low	Low	Low	Low	High	High
BAT-26	−	−	−	−	+	+
NR-21	−	+	−	−	+	+
BAT-25	−	−	−	+	+	+
MONO-27	−	−	+	−	+	−
NR-24	+	−	−	−	−	−

T, tumor size; N, nodal status; pTN, pathological tumor and nodal stage

## Discussion

The purpose of this study was to assess MSI in Japanese female TNBCs to determine the potential use of ICIs for treatment. We demonstrated that the frequency of MSI-H was 0.9% (2/228) in a relatively large cohort. This result was similar to previous reports that included a small cohort of TNBCs [7, 22, 23]. Furthermore, our results suggested that MSI was not common in TNBCs, although those with MSI-H might benefit from ICIs.

In this study, four tumors with MSI-L and two with MSI-H were all basal-like. Basal-like breast cancers constitute one of five intrinsic subgroups of breast cancer, which were determined by microarray-based expression profiling studies [24]. These tumors are often referred to as TNBCs, because most basal-like tumors are typically negative for ER, PR, and HER2 [25]. Basal-like tumors showed a high frequency of *TP53* mutations (80%), indicating a loss of *TP53* function is characteristic for most basal-like cancers. In addition to the loss of *TP53*, MEMo analysis revealed that the loss of *RB1* and *BRCA1* are also basal-like features [25]. Although these genetic mutations in TNBCs are common and specific compared with other subtypes, they have not been established as biomarkers for treatment strategies to date. In the previous study, we assessed BRCAness in TNBCs and showed significantly NG3, high Ki67 and basal-like in TNBCs with BRCAness [17]. However, in this study, two tumors with MSI-L only had BRCAness and two tumors with MSI-H had non-BRCAness despite basal-like features. Further investigations are needed.

MSI is a landmark of genetic instability characterized by frequent errors occurring during the replication of short nucleotide repeats [23]. Testing colorectal cancers for MSI is an effective method to screen for LS, because 90% of LS show MSI-H [26]. LS is characterized by the development of neoplastic lesions in endometrial, gastric, renal, ovarian, and skin tissues [27, 28]. None of six patients with MSI has those tumors in the present study. The 1997 Bethesda guidelines recommend a reference panel of five microsatellites (“Bethesda panel”) for testing: two mononucleotide loci (BAT-25 and BAT-26) and three dinucleotide loci (D2S123, D5S346, and D7S250). The Promega Corporation (Madison, WI, USA) has developed a widely used alternative to the Bethesda panel, called the MSI Analysis System, which replaces the dinucleotide markers with mononucleotide markers (NR-21, NR-24 and MONO-27) [6, 29–31]. These five microsatellite markers have a longer target loci and better sensitivity than the dinucleotide markers. From now on, MSI will be examined with NGS such as various multiplex gene sequencing tests, including tumor mutational burden simultaneously.

Some studies reported an association between breast cancer and MSI. A previous report showed a correlation between the presence of MSI and the absence of both ER and PR [32]. In this report, MSI was detected using PCR at 10 microsatellite markers that were selected to include mono- and dinucleotides and to represent different chromosomes, some of which have been involved in LOH or linked to familial breast cancer. Six of 88 breast cancers (7%) showed MSI, and then four of six had ER- and PgR negative features. However, it is difficult to determine the features of MSI breast cancer, because MSI is remarkably rare in breast cancer [33, 34]. A study reported that in 267 breast cancers, no tumors had MSI using PCR at 104 primers, including markers considered to be highly reliable for detection of MSI in colorectal cancers and reported previously to have in breast cancers [7]. Moreover, the frequencies and characteristics of MSI breast cancer have not been evaluated by subtypes. This study is the first to report the MSI in TNBCs analyzed using the five recommended microsatellite markers without a normal tissue control. We found that the frequency of MSI-H was very rare, but present even in TNBCs.

Recently, the mechanism involved in immune responses in the cancer microenvironment has attracted attention. A previous report suggested that tumors with high Th1/cytotoxic T lymphocyte infiltration had defects in MMR, resulting in MSI, and the increased mutational burden in tumors with MSI created neoantigens related to the immune response, and the immune checkpoint proteins including PD-1 and PD-L1 were upregulated to enable their survival [35]. In our previous study, we focused on T-box transcription factor 21 (T-bet), which is the master regulator of effector T-cell activation, and showed significant relationships among NG3, high Ki67, PD-L1 positivity on tumor cells and CD8 positivity on immune cells in TNBCs with high T-bet-expressing TILs [21]. However, in this study, we were unable to find the consistent results regarding TILs, CD8 and PD-L1 despite NG3 and high Ki67 labeling index. A follow-up clinical trial demonstrated the utility of MSI status as a predictive marker for responses to PD-1 blockade and survival in stage IV cancer patients with dMMR colorectal and non-colorectal cancer [2]. In addition, the PD-1/PD-L1 blockade had an acceptable safety and antitumor activity for TNBCs in the phase Ib KEYNOTE-012 Study [36]. Meanwhile, the IMpassion130 trial revealed that atezolizumab showed efficacy in advanced TNBCs with PD-L1 expressing immune cells [37]. Therefore, ICIs are expected to improve survival in breast cancer patients with MSI-H and/or dMMR.

This study had some limitations. First, the cohort was collected retrospectively. Second, although we assessed MSI, the relationship between MSI and ICIs in TNBCs remains unclear, because ICIs were just started to be used for patients with breast cancer in Japan. Our final goal is to identify specific biomarkers for TNBCs, which may predict the treatment effect or resistance for ICIs.

In conclusion, our results demonstrated that MSI-H might be uncommon. However, true targets for ICIs were present in 0.9% of TNBCs, whose features were not identified by other biological characteristics. We thought it was essential to investigate the frequency of biomarkers such as MSI and TMB further to determine the use of ICIs for TNBC treatment.

## Abbreviations

TNBCs: Triple-negative breast cancers
ER: Estrogen receptor
PR: Progesterone receptor
HER2: Human epidermal growth factor receptor 2
MSI: Microsatellite instability
MMR: Mismatch repair
dMMR: Mismatch repair deficiency
LS: Lynch syndrome
PD-1: Programmed cell death 1
PD-L1: Programmed death-ligand 1
pMMR: Mismatch repair-proficient
FDA: Food and Drug Administration
ICI: Immune checkpoint inhibitor
MSI-H: High-frequency microsatellite instability
CDx: Companion diagnostic
TMB: Tumor mutational burden
NGS: Next-generation sequencing
PCR: Polymerase chain reaction
IHC: Immunohistochemistry
FFPE: Formalin-fixed paraffin-embedded
QMVR: Quasi-monomorphic variation range
MSI-L: Low-frequency microsatellite instability
MSS: Microsatellite stable
MLPA: Multiplex ligation-dependent probe amplification
TILs: Tumor-infiltrating lymphocytes
HE: Hematoxylin and eosin
EGFR: Epidermal growth factor receptor
CK5/6: Cytokeratin 5/6
NG: Nuclear grade
T-bet: T-box transcription factor 21
